# A Complex Radiomic Signature in Luminal Breast Cancer from a Weighted Statistical Framework: A Pilot Study

**DOI:** 10.3390/diagnostics12020499

**Published:** 2022-02-15

**Authors:** Rossana Castaldo, Nunzia Garbino, Carlo Cavaliere, Mariarosaria Incoronato, Luca Basso, Renato Cuocolo, Leonardo Pace, Marco Salvatore, Monica Franzese, Emanuele Nicolai

**Affiliations:** 1 IRCCS Synlab SDN, Via E. Gianturco, 113, 80143 Naples, Italy; rossana.castaldo@synlab.it (R.C.); nunzia.garbino@synlab.it (N.G.); carlo.cavaliere@synlab.it (C.C.); mariarosaria.incoronato@synlab.it (M.I.); luca.basso@synlab.it (L.B.); direzionescientifica.irccssdn@synlab.it (M.S.); emanuele.nicolai@synlab.it (E.N.); 2 Department of Clinical Medicine and Surgery, University of Naples Federico II, 80138 Naples, Italy; renato.cuocolo@unina.it; 3 Interdepartmental Research Center on Management and Innovation in Healthcare—CIRMIS, University of Naples Federico II, 80138 Naples, Italy; 4 Laboratory of Augmented Reality for Health Monitoring (ARHeMLab), Department of Electrical Engineering and Information Technology, University of Naples Federico II, 80138 Naples, Italy; 5 Department of Medicine, Surgery and Dentistry “Scuola Medica Salernitana”, University of Salerno, 84084 Fisciano, Italy; lpace@unisa.it

**Keywords:** breast cancer, radiomic features, molecular biomarkers, normalization, PCA, machine learning

## Abstract

Radiomics is rapidly advancing in precision diagnostics and cancer treatment. However, there are several challenges that need to be addressed before translation to clinical use. This study presents an ad-hoc weighted statistical framework to explore radiomic biomarkers for a better characterization of the radiogenomic phenotypes in breast cancer. Thirty-six female patients with breast cancer were enrolled in this study. Radiomic features were extracted from MRI and PET imaging techniques for malignant and healthy lesions in each patient. To reduce within-subject bias, the ratio of radiomic features extracted from both lesions was calculated for each patient. Radiomic features were further normalized, comparing the z-score, quantile, and whitening normalization methods to reduce between-subjects bias. After feature reduction by Spearman’s correlation, a methodological approach based on a principal component analysis (PCA) was applied. The results were compared and validated on twenty-seven patients to investigate the tumor grade, Ki-67 index, and molecular cancer subtypes using classification methods (LogitBoost, random forest, and linear discriminant analysis). The classification techniques achieved high area-under-the-curve values with one PC that was calculated by normalizing the radiomic features via the quantile method. This pilot study helped us to establish a robust framework of analysis to generate a combined radiomic signature, which may lead to more precise breast cancer prognosis.

## 1. Introduction

Recently, radiomics has been widely used in tumor research. The enormous advantage of radiomics is the automatic extraction of high-dimensional features from digitally encrypted medical images that hold information related to tumor pathophysiology, which can later be mined and analyzed for decision support [1,2,3,4]. 

Radiomics can support the characterization of tumor heterogeneity from macroscopic images and may also provide insights in precision medicine related to tumor detection and subtype classification along with molecular analyses [4,5,6,7]. 

Breast cancer is the most common malignant tumor in females [8]. Breast cancer can be classified into molecular subtypes: (1) luminal-like, (2) Erb-B2+ (human epidermal growth factor receptor 2 [HER2]-enriched), and (3) basal-like, according to Perou et al. [9]. Luminal-like tumors are the most common type of breast cancer, and they can be subclassified into two subgroups, Luminal A and B, based also on the level of Ki-67 expression. Ki-67 has been identified as a molecular marker for the effective assessment of the cell proliferation index [10]. In the same way, the histologic grade (grades 1, 2, and 3) is used to determine the aggressiveness of a tumor. It provides prognostic information in many tumors, including breast cancer [11]. 

In this study, we focused our attention on the luminal A and luminal B subtypes of breast cancer. Several studies have also explored the use of radiomics to investigate Luminal A and B breast cancer patients [12,13,14]. 

In fact, radiomics is a non-invasive method that could provide characterization of tumors. On the other hand, the radiomic analysis workflow still needs to be improved in order to overcome several problems for the construction of robust and reliable radiomic signatures and models to be transferred into clinical practice for the purposes of prognosis, disease tracking, and the evaluation of disease response to treatment [15]. For instance, the radiomic signature is sensitive to variation in the medical images used in radiomic analysis in regard to image quantification and post-extraction feature normalization. 

An important and often undervalued aspect in the radiomic framework of analysis is, in fact, post-extraction feature normalization. Normalization standards are needed for quantitative radiomic features to reduce the within-subject bias effect that affects the comparison of different radiomic features in a single patient (differences because of the conditions of interest) and the between-bias effect that alters the comparison of the radiomic features among patients (namely technical effects, due to their basic differences of scale, range, and statistical distributions). Untransformed features may have high levels of skewness, which can result in artificially low p-values in statistical analyses and eventually introduce bias into developed models [16]. Errors in normalization can have a significant impact on downstream analysis, such as inflated false positives.

In the existing literature, guidelines and precise criteria designed to be used consistently to normalize quantitative radiomic features seem to be missing [17]. On the other side, several efforts have been shown to improve the normalization procedures for image quality as a crucial pre-processing step to correct the imaging-related batch effects before extracting quantitative radiomic features [15,18,19,20,21]. The most common image pre-processing for the texture analysis approaches are the limitation of dynamics to µ ± 3σ (where µ is the mean gray-level value and σ is the standard deviation) and gray level compression based on the range between δ and 2δ (where δ is the number of bits per pixel), among others [22,23]. Although image pre-processing normalization is decisive to reduce the technical variability across images, additional feature normalization steps are still needed during post-processing to reduce the within-subject bias and between-subjects bias effects and, in the case of quantitative features coming from a multicenter study, to identify a batch-specific transformation to express all the data in a common space. Therefore, it is crucial to understand how normalization methods can impact on downstream analysis, such as feature reduction, statistical analysis, and classification problems. 

In radiomics, there are different methods of dimensionality reduction and feature selection [24]. Principal component analysis (PCA) is a well-known approach and one of the most-used methods for feature reduction [25,26], although other methods for supervised feature selection, such as LASSO, have been widely used in radiomic studies. PCA aims to create a smaller set of maximally uncorrelated variables from a large set of correlated variables and to explain as much of the total variation in the data set as possible with the fewest possible principal components (PCs) [27,28].The PCs are linear combinations of features that are ordered by the amount of total variance they explain. The first PCs represent the predominant pattern in the data [28,29]. Normalization methods can help features arrive in a more digestible form for these algorithms by making every feature in proportion with each other, otherwise they will tend to perform poorly [30]. In this study, PCA was firstly chosen as an explorative tool to visualize how the data normalization methods are able to disclose different aspects of the data in the scores and the accompanying loadings. Furthermore, it allows the identification of the most important radiomic features for the characterization of breast cancer by analyzing the loadings in order to generate a combined radiomic signature [31].

The main aim of this study was to investigate the effect of within-subject and between-subjects normalization methods on the PCA and downstream analysis. In order to address this aim, we designed an ad hoc weighted statistical framework to investigate how different normalization techniques could impact on the PCA and data analysis to explore radiomic signature in breast cancer. 

Thus, the main goal of this study is to provide a multivariate statistical framework via PCA to generate a complex quantitative radiomic signature, which may lead to more precise breast cancer prognosis and help clinicians in decision-making towards personalized medicine.

## 2. Materials and Methods

### 2.1. Patient Selection

The study was approved by the institutional ethics committee in accordance with the ethical guidelines of the 1975 Declaration of Helsinki and approved by the ethical committee of the institution “IRCCS Synlab SDN” (Protocol no.2-11). All subjects included in the study provided informed consent. The recruitment of the patients took place at IRCCS Synlab SDN. During the period of 2011 to 2014, consecutive patients were enrolled in the study. The inclusion criteria were (a) a diagnosis of breast cancer confirmed by an immunohistochemistry (IHC) report, (b) the absence of any prior surgical or pharmacological treatment for breast cancer (naïve), (c) a negative previous personal oncological history, (d) >18 years of age, and (e) lesions of at least 0.2 cm for a comprehensive imaging characterization.

Exclusion criteria were patients with (a) pregnancy; (b) blood glucose levels greater than 140 mg/dL (7.77 mmol/L); (c) inadequate PET images, MR images, or both, due to artifacts, system malfunction, or poor patient cooperation; (d) contraindication to MR imaging; and (e) inability to tolerate being in the PET or MR imaging apparatus. After all these exclusion criteria, 36 patients were included in the study.

### 2.2. Clinical Parameters

Among the 36 enrolled patients, clinical parameters were available for 27 female patients. All clinical characteristics, such as age, tumor size, tumor locations, number of lesions, and tumor subtype, were recorded for each patient. The tumor size in each enrolled patient was calculated on maximum intensity projection (MIP) of subtraction post-contrast images [32]. One senior radiologist that was experienced in breast imaging (more than 15 years of experience) and one nuclear medicine specialist (more than 20 years of experience) reviewed the local tumor size, tumor locations, number of lesions, and tumor staging in consensus.

Estrogen, progesterone, and HER2 receptor status were reported, along with the tumor molecular subtype classification, cellular differentiation status, and proliferation index (Ki-67) of the tumor lesions using the immunohistochemical (IHC) information of the enrolled BC patients, if present.

### 2.3. Circulating miRNA Signatures

Quantification of miRNAs expression values using the miScript miRNA PCR Array and validation by real-time PCR (qRT-PCR) were performed according to the protocol proposed by M. Incoronato, et al. [33]. The relative expression for each miRNA was calculated as 2^−∆CT^ and from a differential expression analysis to discriminate the breast cancer condition with respect to healthy status [33]. In total, 5 (miR-125b-5p, miR-143-3p, miR-145-5p, miR-100-5p, and miR-23a-3p) out of the 84 miRNAs processed were differentially expressed and upregulated in the plasma samples of the breast cancer patients with a fold change ≥1.5 and a *p*-value < 0.05. Therefore, only those five miRNAs were considered in the following analysis to evaluate, if present, possible associations with radiomic signatures combined with clinical parameters.

### 2.4. Image Acquisition and Pre-Processing

PET/MR was performed on a 3T Biograph mMR (Siemens Healthcare, Erlangen, Germany). Bed position was established in order to obtain a full coverage of the breast region using a dedicated breast coil. Each patient was positioned prone and feet first, paying attention to correctly position the breast inside the dedicated coil cavities. Through a venous access, the patient was connected to an automatic injector useful for the administration of the contrast gadolinium diethylene triamine pentaacetate agent (Gd-DTPA; Magnevist, Bayer Inc., Mississauga, ON, Canada) at 0.1 mmol/kg body weight and a flow rate of 3.5 mL/s. For PET, all patients fasted for at least 6 h before the procedure. They then received 400 ± 32 MBq (mean ± standard deviation) of 2-Deoxy-2-[18F] fluoroglucose(18F-FDG) intravenously. After a biodistribution period of 60 min and before breast PET/MR, all the patients underwent total body PET/CT. Subsequently, a simultaneous PET/MR scan was acquired. The PET/MRI sequences taken into account for this study were: a PET acquisition of 8 min; an axial T2-weighted half Fourier single-shot turbo spin echo (HASTE) (TR 1400 ms, TE 89 ms, slice thickness 6 mm, FOV 399 × 399 mm, acquisition matrix 384); an axial diffusion-weighted imaging (DWI) with b values of 50, 400 and 800 s/mm^2^ (TR 7000 ms, TE 83 ms, slice thickness 4 mm, FOV 223 × 400 mm, acquisition matrix 190), with automatic apparent diffusion coefficient (ADC) map reconstruction; an axial high-resolution T1-weighted VIBE sequence with fat suppression was acquired after contrast agent injection (TR 8.69 ms, TE 4.33 ms, slice thickness 0.9, FOV 337 × 360 mm, acquisition matrix 192). The PET data were reconstructed with an AW OSEM 3D iterative reconstruction algorithm applied with 3 iterations and 21 subsets and Gaussian smoothing of 4 mm in full width at half maximum. MR attenuation correction was performed via a segmentation approach based on 2-point Dixon MRI sequences.

### 2.5. Radiomic Features

Radiomic features were extracted from 36 patients with PMOD, an automated quantitative software of images in biomedical research (version 3.8, PMOD Technologies Ltd., Zürich, Switzerland). Quantitative imaging was performed by the PMOD tool PBAS, performing a semi-automatic segmentation of breast lesions using a VOI isocontour. The semi-automatic segmentation of breast lesions was carefully supervised by an expert radiologist with more than 10 years of experience.

For this study, PET images and the MR sequences T2w HASTE, ADC, and T1w were analyzed post-contrast-injection after a 3D rigid registration with a normalized mutual information on PMOD. Firstly, the isocontour VOI was placed on the tumor lesion on PET acquisition by an experienced nuclear medicine physician, considering a metabolic tumor volume (MTV) with a threshold of 40% of the maximum signal intensity (MTV40) for a volumetric characterization of lesion burden [34]. Then, the VOI was entirely copied on the morphological T2w, ADC, and T1w sequences, verifying its coverage on the axial, sagittal, and coronal planes. Furthermore, for each patient and sequence, contralateral healthy gland tissue information was obtained by translating the same VOI isocontour produced previously in the same contralateral breast quadrant. For each VOI, the texture-analysis parameters were extrapolated using an integrated PMOD software. An example of an 18F-FDG-PET/MRI scan image is presented in Figure S1. Imaging normalization and resampling were applied during pre-processing using the default parameters of the PMOD software.

A total of 74 radiomic features were extracted from the PET/MR imaging with the 3D extraction. In total, 24 radiomic features were extracted from PET, including 5 first-order features from the intensity histogram computed on 256 bins (with a bin size of 32), namely, mean, variance, skewness, kurtosis, and energy, and 16 features from the SUV analysis.

From the MRI images, 5 first-order features were extracted from the intensity histogram computed on 256 bins for each of the ADC, T2w, and T1w post-contrast sequences (mean, variance, skewness, kurtosis, and energy). In addition, 19 second-order features were also computed for the both the T2w and T1w post-contrast images [26,27], including energy, contrast, entropy, homogeneity, correlation, sum average, variance, dissimilarity, and autocorrelation. 

A summary of the extracted radiomics features is presented in Table S1.

### 2.6. Statistical Analysis

A statistical analysis was performed using R software (version 3.6.1, Vienna, Austria) [31]. Continuous variables were expressed as means, standard deviations (SD), medians and ranges. The data were tested for normality through the Shapiro–Wilk test. Radiomics data were tested for normality before and after applying the normalization methods. The Wilcoxon rank-sum test or *t*-test were used, as required, for comparisons between groups. Categorical variables were expressed as percentages and were compared using the chi-square test or Fisher’s exact test. A *p*-value less than 0.05 was considered significant. Holm’s correction was used for multiple hypothesis correction, if necessary. Spearman’s rank correlation was carried out for continuous variables. A Spearman’s ρ value greater than 0.8 and a significant *p*-value (*p*-value < 0.05) were set as the threshold to identify a strong agreement between variables. As a rule of thumb, a Spearman’s correlation ρ value that lies between 0.80 and 1.00 is considered to identify a strong correlation among the variables, as also reported in [35].

#### 2.6.1. Radiomic Statistical Analysis

Within-subject normalization was achieved as the ratio between the radiomic features extracted from the malignant and healthy breasts for each of the 36 patients enrolled in this study. Successively, three different normalization techniques were, used to normalize the radiomic features to also reduce between-subjects bias. The features were normalized using z-score normalization, where each feature was normalized as z = (x − $\bar{x}$)/s, where x, $\bar{x}$, and s are the feature, the mean, and the standard deviation, respectively [36]. Quantile normalization, which transforms the original data to remove unwanted technical variation by forcing the observed distributions to be the same, and the average distribution, obtained by taking the average of each quantile across samples, is used as the reference [37,38]. Lastly, whitening normalization was used. This methos is based on a linear transformation that converts a vector of random variables with a known covariance matrix into a set of new variables whose covariance is the identity matrix, meaning that they are uncorrelated and each have a variance equal to one [39]. The radiomic features were normalized separately for each MRI sequence and PET [40].

**Figure 1:**
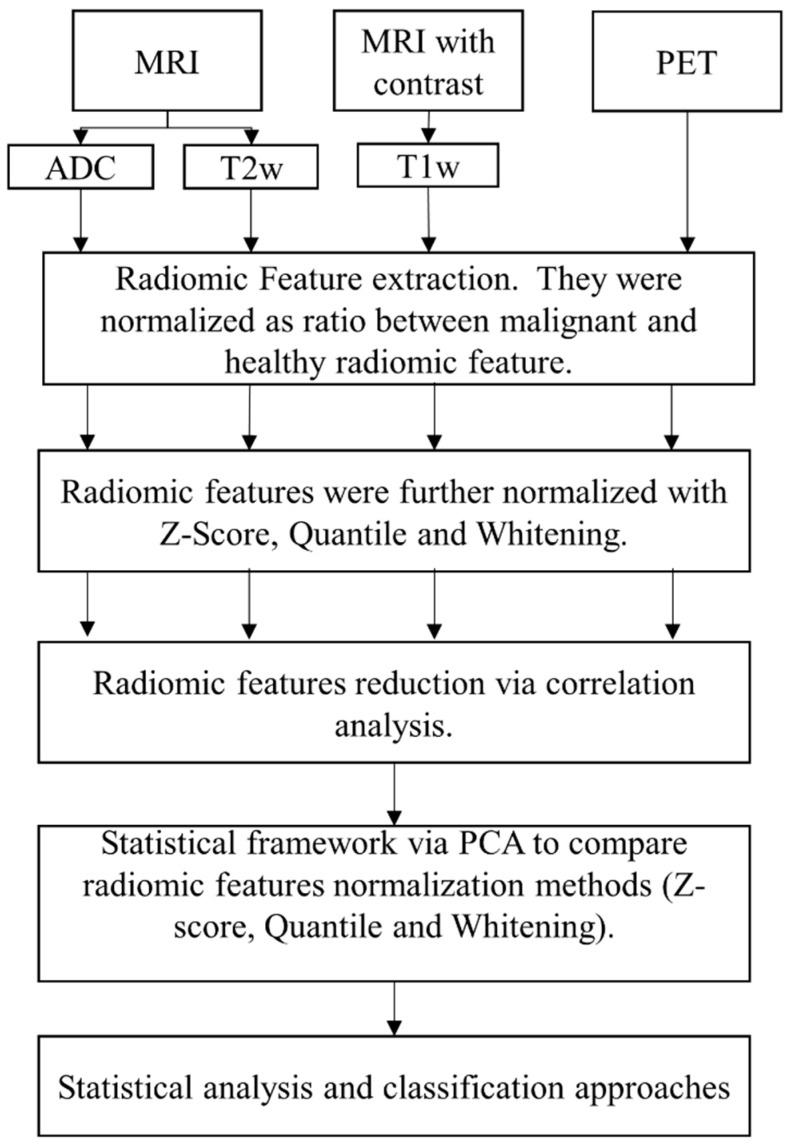
Analysis Workflow.

After the normalization step, normalized radiomic features, extracted from different imaging techniques, were reduced separately by excluding highly correlated features via Spearman’s correlation with a ρ value greater than 0.8 and significant *p*-values (*p*-value < 0.05). The absolute values of the pair-wise correlations were considered. If two variables had a high correlation, we looked at the mean absolute correlation of each variable and removed the variable with the largest mean absolute correlation [41]. Normalized and reduced radiomic features, extracted from different imaging techniques, were then merged into one dataset. A principal component analysis was applied to a merged, normalized, and uncorrelated dataset [42]. The analysis pipeline is shown in Figure 1. 

#### 2.6.2. PCA on Radiomic Features

A PCA was applied to radiomic features in four datasets:

1. Radiomic features normalized as the ratio of malignant and healthy radiomic features.
2. Radiomic features normalized as the ratio of malignant and healthy radiomic features and z-scores.
3. Radiomic features normalized as the ratio of malignant and healthy radiomic features and quantiles.
4. Radiomic features normalized as the ratio of malignant and healthy radiomic features and whitening.

**Figure 2:**
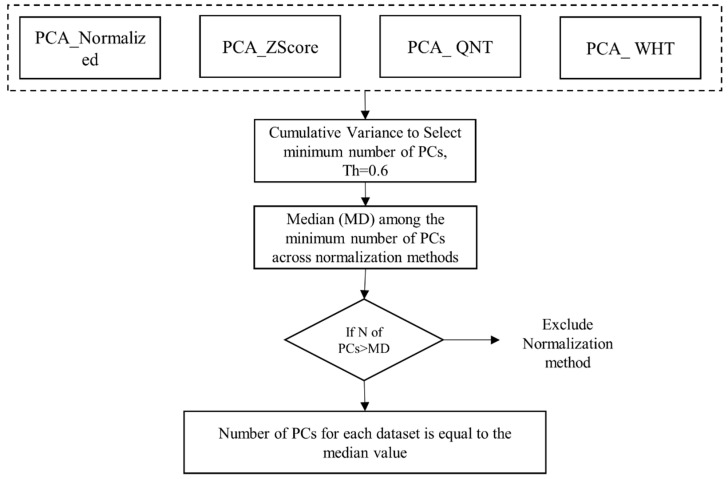
Workflow for PC reduction. PCA: principal component analysis; QNT: quantile normalization method; WHT: whitening normalization method; PCs: principal components; Th: threshold; MD: median.

Cumulative variance was set to 60% to select the minimum number of PCs. In order to only select the normalization methods that were able to better explain the variance in the data via PCA, the median value was computed among the number of PCs that explained 60% of the variance across datasets (1 to 4). The normalization methods that had more PCs compared to the median value were excluded from further analysis (Figure 2). The number of PCs across all datasets was set equal to the median value. 

The loading and variable contributions for all datasets were explored. For each dataset, the third quantile of the distribution values of the loadings was chosen as the threshold to identify a strong effect on the principal components. Positive loadings indicate that a variable and a principal component are positively correlated (e.g., an increase in one leads to an increase in the other). Negative loadings indicate a negative correlation.

#### 2.6.3. Clinical Investigation and Patient Stratification

Statistical analyses were performed to explore the normalization impacts on the PCs and radiomic features to investigate the grade, the Ki-67 index characterizing the aggressiveness of the tumor, and the tumor subtype. Due to missing data, clinical investigations were carried out in 27 female patients. 

Firstly, patients that presented with grade 2 tumors were divided from the patients that presented with grade 3 tumors. Therefore, patients were stratified by tumor grade in two classes: G2 and G3. None of the included patients presented with G1 tumors.

Secondly, the population was stratified into two classes to compare tumor conditions based on the nuclear protein Ki-67, which is considered a good indicator of cellular proliferation. The threshold value was fixed to 30% [43,44]. Therefore, patients presenting values of Ki-67 greater than 30% were included in Class 1 (i.e., high values of Ki-67), whereas patients presenting values of Ki-67 less than 30% were included in Class 2 (i.e., low values of Ki-67).

Thirdly, patients that presented with the luminal A tumor type were divided from the patients that presented with the luminal B tumor type. Therefore, patients were stratified by tumor subtype into two classes: Luminal A, Luminal B. HER2 (+) tumor cases were excluded at this stage due to the very low number of patients.

#### 2.6.4. Classification Methods

The classification approaches were considered to automatically classify the tumor grade, the tumor condition stratified via Ki-67, and the tumor subtype, based on statistically significant PCs and the radiomic features that contributed the most to the statically significant PCs and clinical characteristics. The classification approaches were investigated to empirically understand the impact of the data normalizations and PCA. R software (version 3.6.1, Vienna, Austria) [31] was used to develop the classifiers.

Three traditional classification methods were investigated: additive logistic regression (LogitBoost), which is a boosting algorithm as an approximation to additive modelling on the logistic scale using the maximum Bernoulli likelihood as a criterion [45]; random forest decision trees (RF), an ensemble learning method for classification that operates by constructing a multitude of decision trees during training and outputting the class that is the mode of the classes (classification) [46,47]; and linear discriminant analysis (LDA), which consists of finding the projection hyperplane that minimizes the interclass variance and maximizes the distance between the projected means of the classes [48]. These classification methods were chosen because of the nature of the datasets (i.e., small dataset) and according to the existing literature. In fact, LogitBoost, decision trees, and LDA have been widely used in breast cancer detection [24,49].

For LogistBoost and RF, we employed the default configuration provided in RStudio [41]. For LogistBoost, the number of boosting iterations was set to 100. In the random forest analysis, the number of available variables for splitting at each tree node was calculated as the square root of the number of predictor variables (rounded down). The repeated 3-fold stratified cross-validation approach was used to validate the models [50,51]. Due to the unbalanced nature of the dataset, the SMOTE technique was used to attenuate the bias towards the classification in the majority class in each training fold [52]. Repeated cross-validation was performed to guarantee the robustness of the results and to reduce overfitting [53]. Cross-validation was repeated 100 times. Binary classification performance measures were adopted according to standard formulae [54].

Due to the low number of patients included in the study, no more than 1 feature for every 10 “observation/subject” presenting the outcome of interest was employed to develop the models, as described in [55]. The models were trained and validated by using relevant statistical features (*p*-value less than 0.05) for the outcome of interest [17]. That means that predictors were evaluated independently before the data were applied to the classification methods, as described in [24]. In fact, Zerouaoui et al. reported that the majority (47%) of the studies in the field of radiomic in breast cancer have used a priori feature selection via filter methods. 

Among the three different methods (LogitBoost, RF, and LDA) used to train and validate the classifiers, the best performing method was chosen as the one achieving the highest value of sensitivity + specificity [56]. In fact, as a rule of thumb, this criterion can help interpret the evidence on test performance. For a diagnostic or clinical test to be useful, sensitivity + specificity should be around 1.5 (halfway between 1, which is useless, and 2, which is perfect) [56]. In the case of an equal value of sensitivity + specificity, the model with the highest area under the curve (AUC), which is a reliable estimator of both sensitivity and specificity rates, was considered. 

Comparison analyses among the classification methods were also carried out via a Wilcoxon sum-rank test over 100 repetitions. The values of sensitivity + specificity, Cohen’s Kappa, and AUC were also graphically investigated via boxplots. 

## 3. Results

### 3.1. Study Population

For this study, we enrolled a total of 36 female patients who underwent MRI and PET. For 27 of them we reported the available clinical and molecular characteristics in Table 1. The breast cancer tumor molecular subtypes were classified according to the 2013 St. Gallen guidelines [57]. 

### 3.2. Radiomic Statistical Framework: Normalization and PCA

The majority of the radiomic features (≥86%) before and after normalization (z-score and quantile methods), extracted from 36 female patients, were non-normally distributed via the Shapiro–Wilk test. For the radiomic features normalized via whitening methods, 60% were non-normally distributed. 

The radiomic features were first normalized as the ratio between the malignant features and healthy features. Successively, the radiomic features for each MRI sequence and PET were normalized separately using the z-score, quantile, and whitening normalization methods. The goal of normalizing the feature separately for each dataset is to remove the feature variability between the datasets [40]. 

Spearman’s rank correlation was used to exclude highly correlated features with a threshold of 0.8 separately for each of the MRI and PET sequences. A smaller subset of features was identified and PCA was applied to four datasets:

1. Radiomic features only normalized as the ratio of malignant and healthy radiomic features.
2. Radiomic features normalized as the ratio of malignant and healthy radiomic features and z-scores.
3. Radiomic features normalized as the ratio of malignant and healthy radiomic features and quantiles.
4. Radiomic features normalized as the ratio of malignant and healthy radiomic features and whitening.

**Figure 3:**
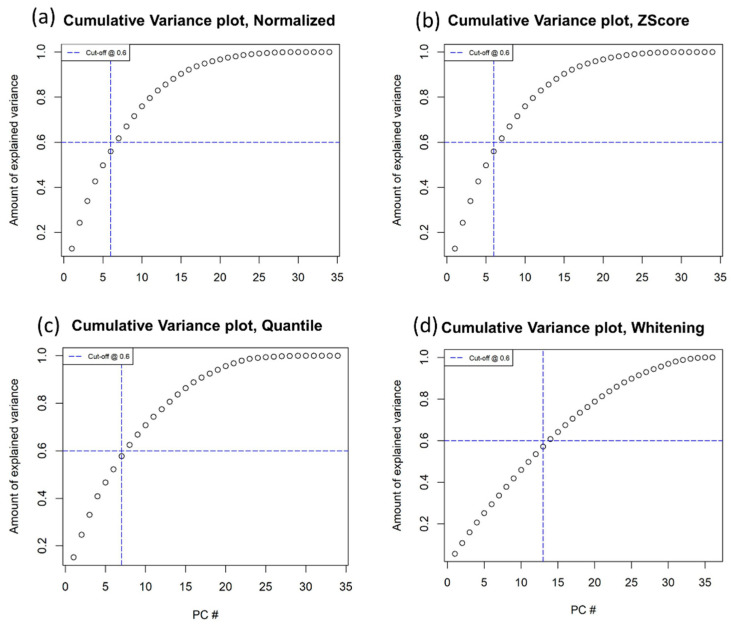
Cumulative variance plots for the 4 datasets, based on a threshold of 0.6. (**a**) Normalized only as the ratio of malignant and healthy radiomic features; (**b**) Z-Score; (**c**) quantile; and (**d**) whitening.

The results showed that 6 PCs explained 60% of the total variance in the case of within-subject normalization and z-score (Figure 3a,b), 7 PCs explained 60% of the total variance using quantile methods (Figure 3c) and 13 PCs explained 60% of the total variance in the whitening methods (Figure 3d). The median among these PCs was seven. As shown in Figure 3, in the WHT normalization method, more than 7 PCs explained 60% of the total variance. This was a reason to exclude this normalization method from the other analyses. Therefore, to harmonize the number of PCs across the datasets, seven PCs were considered. The loadings of the three datasets were explored, as shown in Figure S2. 

### 3.3. Clinical Investigation and Classification Approaches

#### 3.3.1. Grade

**Figure 4:**
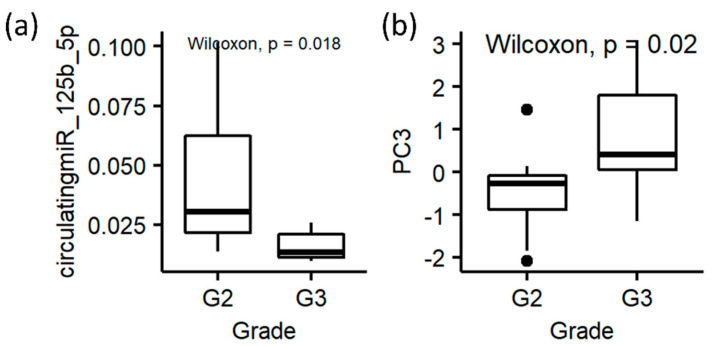
Box plots for tumor grade (i.e., G2 and G3). (**a**) Circulating miRNA_125b_5p; (**b**) PC3 from the quantile dataset.

Among the 19 patients that reported tumor grading, 11 were grade 2 and 8 patients were classified as grade 3. None of the patients included in the study presented with grade 1 tumors. From the statistical analysis, the circulating miRNA 125b_5p and PC3 from the quantile normalization dataset showed to be significantly different between the patients with grade 2 and grade 3 tumors (Figure 4). 

**Figure 5:**
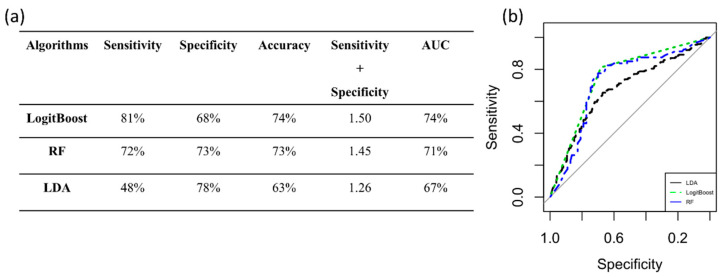
Performance measures to classify tumor grade. (**a**) Algorithm performance via PC3 to classify tumor grade. (**b**) ROC curves of the three classifiers. AUC: area under the curve; RF: random forest; LDA: linear discriminant analysis.

The classification algorithms were trained and validated using PC3 to automatically classify tumor grade (grade 2 and grade 3). The performance measures are reported in Figure 5a. The best classifier, with a value of 1.5 for Sensitivity+ Specificity and an AUC of 74%, was LogitBoost, as shown in Figure 5b.

LogitBoost was shown to outperform the other models, as shown in Figure S3. In particular, the Sensitivity + Specificty and Kappa values were statistically (*p*-value less than 0.05) higher in LogistBoost (LB) than in LDA.

Moreover, we investigated the variables that contributed the most to PC3 (via QNT normalization), as shown in Figure S4. The LogitBoost algorithm was also trained and validated using the first two variables (toa.bw and glcm_information_correlation_1_with_contrast_agent) to investigate tumor grade. LB achieved a sensitivity, specificity, accuracy, and AUC of 53%, 68%, 61%, and 63%, respectively. However, the performance was lower than the performance achieved using PC3.

#### 3.3.2. Ki-67

**Figure 6:**
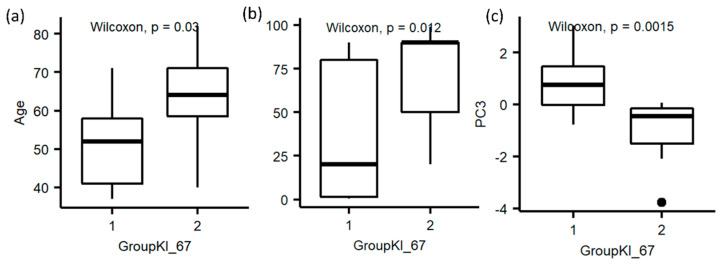
Box plots for high and low values of Ki-67 (i.e., Class 1 and 2). (**a**) Age; (**b**) Progesterone status; (**c**) PC3 from the quantile dataset.

Among the 24 patients that reported Ki-67 values, 11 had a value of Ki-67 greater than 30% (Class 1) and 13 had a value less than 30% (Class 2). From the statistical analysis, age, progesterone receptor status, and PC3 from the quantile normalization dataset were significantly different between the patients with high and low values of Ki-67 (Figure 6).

**Figure 7:**
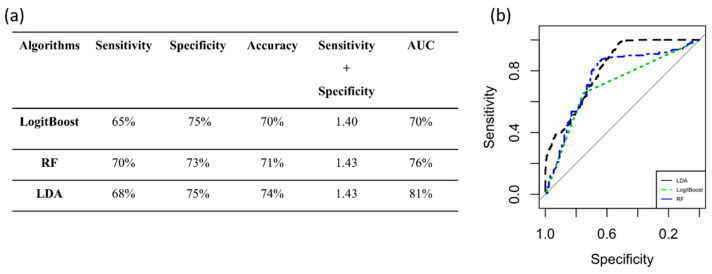
Performance measures to classify high and low values of Ki-67. (**a**) Algorithm performance via PC3 to classify high and low values of Ki-67. (**b**) ROC curves of the three classifiers. AUC: area under the curve; RF: random forest; LDA: linear discriminant analysis.

The classification algorithms were trained and validated using PC3 to automatically classify high and low values of Ki-67. The performance measures are reported in Figure 7a. The best classifier, with a value of 1.43 for Sensitivity + Specificity and an AUC of 81%, was LDA, as shown in Figure 7b.

LDA outperformed the other models, as shown in Figure S5. In particular, the area under the ROC curve was significantly higher (*p*-value less than 0.001) in LDA than in LB.

The LDA algorithm was also trained and validated using the first three variables (toa.bw and glcm_information_correlation_1_with_contrast_agent) to investigate values of Ki-67. LDA achieved a sensitivity, specificity, accuracy, and AUC of 61%, 56%, 58%, and 60%, respectively. However, the performance was significantly lower than the performance achieved using PC3.

#### 3.3.3. Luminal A and B

Among the 23 patients that presented with molecular subtypes, 18 were Luminal B and 5 were Luminal A.

**Figure 8:**
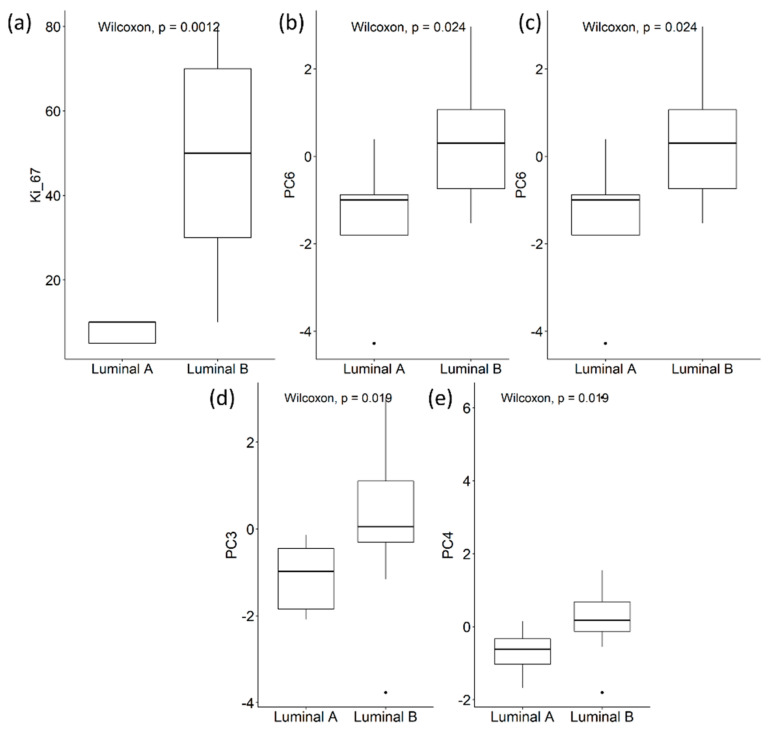
Box Plots for the tumor subtypes (i.e., Luminal A and Luminal B). (**a**) Ki-67; (**b**) PC6 from the non-normalized dataset; (**c**) PC6 from z-score dataset; (**d**) PC3 from the quantile dataset; (**e**) PC4 from the quantile dataset.

From the statistical analysis, Ki-67, PC6 from the non-normalization and z-score datasets and PC3 and PC4 from the quantile normalization dataset were statistically significant different between patients presenting with Luminal A- and B-type tumors (Figure 8). The classification algorithms were trained and validated using each principal component that was significantly different between Luminal A and B.

**Figure 9:**
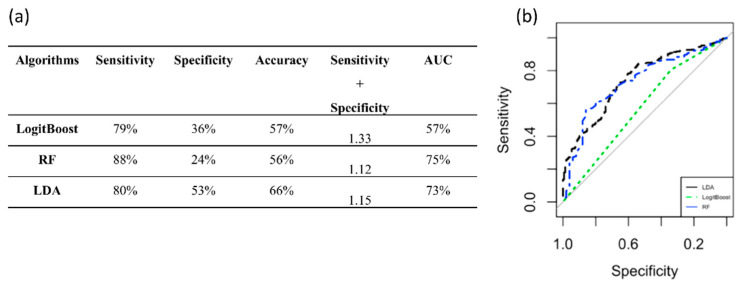
Performance measures to classify the tumor subtypes. (**a**) Algorithm performance via PC3 to classify the tumor subtypes. (**b**) ROC curves of the three classifiers. AUC: area under the curve; RF: random forest; LDA: linear discriminant analysis.

The performance achieved by LDA, RF, and LogitBoost using PC6 from the non-normalized and z-score datasets and PC3 and PC4 from the quantile normalization dataset, are shown separately in Table S2. In terms of the values of sensitivity + specificity, the best classification was considered to be LDA in all cases because both RF and LogitBoost presented very low specificity, although they achieved a high AUC. The LDA method via PC3 from the quantile dataset achieved the highest performance compared to the LDA models developed via PC6 and PC4. Therefore, LDA was able to automatically classify the tumor subtype (Luminal A and B) with a sensitivity + specificity value of 1.33 and an AUC of 73% by only using PC3 normalized via the quantile method (Figure 9b). The performance measures are reported in Figure 9a. RF was not considered to be the best classification even though it presented higher AUC than LDA because it performed very poorly on the detection of Luminal A patients (24% specificity). 

LDA outperformed the other models, as shown in Figure S6. In particular, the area under the ROC curve was significantly higher (*p*-value less than 0.001) in LDA than in LogistBoost (LB). 

The LDA algorithm was also trained and validated using the first two variables (toa.bw and glcm_information_correlation_1_with_contrast_agent) due to the small number of patients included in the analysis. The LDA achieved a sensitivity, specificity, accuracy, and AUC of about 50%. LDA via PC3 showed significantly higher performance than the performances achieved by LDA via radiomic features.

## 4. Discussion

This pilot study established a robust framework of analysis to evaluate quantitative imaging biomarkers and to generate a combined radiomic signature for a more precise breast cancer prognosis, also investigating the effect of within-subject and between-subjects normalization methods on PCA and downstream analysis. 

Several other applications of radiomics in breast cancer imaging have been investigated to differentiate between malignant and benign breast lesions; to predict the axillary lymph node status, molecular subtypes of breast cancer, tumor response to chemotherapy, and survival outcomes; and to discriminate between breast cancers and background parenchymal enhancement [58,59]. However, there are still challenges to be addressed. 

Feature normalization is often an undervalued aspect in the radiomic framework. Data normalization methods are essential for radiomic features, due to their basic differences of scale, range, statistical distributions, and condition of interest. Moreover, the normalization process should produce radiomic features that are replicable, have similar distributions for the same tissues of interest within and across patients, are not influenced by biological abnormality or population heterogeneity, are minimally sensitive to noise and artifacts, and do not result in the loss of information associated with pathology or other phenomena [60].

In this study, in order to reduce the within-subject bias effect that marks the assessment of different radiomic features in a single patient, we normalized the features extracted from the malignant breast with the features extracted from the healthy breast by the calculation a ratio. This is a novel aspect of our study, as several studies have normalized images with respect to healthy tissue, which has also proven to be optimal for other organs, such as the prostate [61], but none of the studies in the existing literature have tried this process on quantitative radiomic features. 

However, it is worth noting that this will not always be possible, since patients often have tumors in both breasts. Nevertheless, if the tumors are not in the same regions, our method is still suitable, as long as the counterpart of the healthy tissue is not affected by the tumor and quantitative radiomic features can be extracted.

To reduce the between-subjects bias effect that alters the comparison of the radiomic features in different patients (namely technical effects, due to their basic differences of scale, range, and statistical distributions), we applied three common normalization methods, also proved to be reliable in [17], z-score, quantile, and whitening normalization methods. Only a few studies have tried to investigate the impact of normalization methods on radiomic features during post-processing [17,62].These studies investigated the effects of different normalization methods on extracted radiomic features to reduce between-subjects bias. Haga et al. [62] investigated CT radiomic features extracted from non-small-cell lung cancer patients. The radiomic features were normalized using the min-max normalization, the z-score normalization, and the whitening methods to improve the accuracy for the histology prediction. The radiomic features that were normalized by z-score achieved the highest AUC value. Castaldo et al. [17] evaluated the effect of several normalization techniques to predict four clinical phenotypes in breast cancer via radiomic features extracted from MRI. This study suggested that the quantitative radiomic analysis is influenced by the normalization method choice. However, in our previous study we did not investigate the effect of within-subject bias along with between-subjects bias on feature reduction methods, such as PCA.

Therefore, we investigated the effect of within-subject and between-subjects normalization methods on the most common feature reduction method, PCA, and evaluated this integrated approach to investigate the molecular cancer subtypes, tumor grade, and Ki-67 proliferation index in breast cancer. After a filtering step, based on a correlation analysis, to remove redundance in the radiomic data, we applied a PCA to generate a complex radiomic signature and investigate the value of performing normalization on extracted radiomic features. 

PCA was applied to different datasets based on the four normalization approaches (i.e., radiomic features only normalized as the ratio of malignant and healthy radiomic features; radiomic features normalized as the ratio of malignant and healthy radiomic features and z-scores; radiomic features normalized as the ratio of malignant and healthy radiomic features and quantiles; and radiomic features normalized as the ratio of malignant and healthy radiomic features and whitening) to investigate the differences among them. By investigating the cumulative variance, we could observe that the case of within-subject normalization provided comparable results with the z-score, and this is reasonable, due to the simplicity of the z-score approach. Slightly different results were achieved for the quantile methods, which needed one more PC compared to the within-subject normalization and z-score. The whitening method was excluded, as it needed 13 PCs to explain 60% of total variance. This was expected, as the whitening method is highly sensitive to outliers [63].

The same radiomic features contributed to the seven PCs for the within-subject normalization and z-score methods, whereas different radiomic features contributed to the PCs for the quantile method. This emphasizes that by applying different normalization methods we could achieve different results, and, therefore, great attention needs to be paid when working with quantitative radiomic features. 

To further investigate the role that normalization methods have on the PCA-based framework, we investigated whether quantitative radiomic features were able to differentiate among the tumor grades, aggressiveness of tumor, and luminal types in females with breast cancer via statistical analysis and classification approaches. 

Tumor grading, together with tumor size and lymph node stage, is often used to stratify individual patients for appropriate therapy. In particular, patients with grade 2 and 3 tumors are referred to as high-risk patients [64]. In this study, we investigated patients presenting with grade 2 and 3 tumors. Regarding the statistical analysis, circulatingmiRNA_125b_5p and the third component (PC3) of the quantile normalization were significantly different among the patients presenting with grade 2 and 3 tumors. LogitBoost achieved an AUC of 74% to automatically identify high-risk patients (tumor grade 3) by using only one PC. 

We also investigated the Ki-67 index, which has recently attracted significant interest from clinical oncologists. In fact, the mitotic index (MI) and the Ki-67 index value are the two most commonly used indices to measure proliferation [65]. Moreover, certain studies reported that the Ki-67 index value is a significant prognostic factor in terms of disease-free and overall survival after initial treatment [66]. In general, high levels of Ki-67 expression in breast cancer correlate strongly with a more tenacious proliferation and a poor prognosis. In this study the cut-off point for Ki-67 was set at 30% as recommended in [43,44]. Therefore, the data were split into low and high values of Ki-67 to assess the aggressiveness of the tumors. Age and progesterone receptor status were significantly higher in patients with high values of Ki-67, wheatears PC3 from the quantile dataset was significantly lower in patients with high values of Ki-67. The best classifier was LDA, which achieved an AUC of 81% to automatically detect patients with a high value of Ki-67 via only PC3. 

Lastly, Luminal A and B were evaluated, as they are the most common tumor subtypes among the worldwide population. Luminal A tumors are more endocrine sensitive, indolent, and have a better prognosis; whereas luminal B are less endocrine sensitive, more aggressive, and have worse prognosis. In fact, it has been demonstrated that luminal B cancers are more progressive, as tumors usually exhibited more nodal metastasis than in luminal A subtype [67,68]. Moreover, the main reason for attempting to distinguish between luminal A and luminal B tumors is because they respond differently to treatment [69]. Regarding the statistical analysis, Ki-67 and the third component (PC3) of the quantile normalization were statically different among subtypes A and B. Ki-67 level was expected to be statistically different, as it is recognized to be a surrogate of “Luminal A-like” disease [68]. The linear discriminant analysis method was also able to differentiate Luminal B with 75% AUC by using only one principial component via the quantile method. This was in line with the recent literature, which states that radiomic assessment of breast imaging can provide an option in determining breast cancer molecular subtypes [14].

The main aim of using the classification methods was to investigate the impacts of normalization methods and the use of PCA on classification performances. A recent study [70] also investigated the impact of 14 data normalization methods as pre-processing steps on classification performance. They observed from the results that no single method outperformed the others on 21 publicly available real and synthetic datasets. According to Singh et al. [70], z-score performed better than non-normalized methods. However, they did not investigate the quantile or whitening methods. 

Overall, the classification methods achieved good results in detecting tumor subtypes, grade, and aggressiveness by using only the third principial component of the radiomic features normalized by the quantile methods. 

The first 10 radiomic features that contributed to PC3 came from the first-order grey-level statistics features (such as energy and skewness) and SUV parameters (the sum of all VOI pixel values) from the PET technique, two features (skewness and variance) came from ADC-MRI, and the majority came from the grey-level co-occurrence matrix-based features from the T1w-MRI. Lastly, only one feature quantifying the complexity of the tumor texture was from the T2-wMRI. This demonstrated that a linear combination of MRI-based and PET-based radiomic features are able to characterize molecular prognostic indicators in females affected by breast cancer. These results are also supported by the recent literature data that indicate that features obtained from PET and MRI correlate with tumor histological characteristics and molecular subtypes [71,72,73,74,75]. Moreover, by using only the first two radiomic features that contributed to PC3, no improvements in the results were shown. In particular, both the accuracy and AUC were significantly (*p*-value < 0.05) higher in the models developed by using only one principal component. This result is worth noting, as, by applying PCA, a combination of features allows a better classification of tumor subtype, grade, and aggressiveness. One of the drawbacks of PCA is that PCs are not as readable and interpretable as the original radiomic features. Conversely, the main advantages of using PCA is to maintain accuracy and make datasets easier to understand. Moreover, using PCA in small dataset can help to generate a quantitative radiomic signature in a composite indicator, where principal components are the linear combination of your radiomic features.

This is particularly relevant when a small dataset is available, as, by rule of thumb, one predictor for every 10 events should be used in classification tasks [17,55]. This result is in line also with Mert et al. [76], who demonstrated that the use of feature reduction methods, such as a pre-processing step to classification analysis, can be a high-performance solution. 

Five circulating miRNAs were selected in this study (miR-125b-5p, miR-143-3p, miR-145-5p, miR-100-5p, and miR-23a-3p) because they showed to be significantly upregulated (*p* < 0.05) in breast cancer patients vs. healthy donors, as reported in [33]. Although they are not all well-accepted biomarkers for breast cancer yet, Incoronato et al. found that the expression levels of miR-125b-5p were variable and depended on the severity of the disease. Additionally, the expression levels of miR-143-3p reached expression values close to those of healthy donors in cancer stage IV. This result suggested that at stage IV, this molecule is not required for the maintenance of the pathology. Regarding miR-145-5p, Tang et al. [77] reported that miR-145-5p played a suppressive role in the proliferation of breast cancer cells and that it is a putative biomarker for risk assessment in patients with breast cancer. Lastly, in vitro functional experiments demonstrated that overexpression of miR-100 inhibited the proliferation, migration, and invasion of breast cancer cells, which suggests that miR-100 may be used as a potential molecular marker and target for the diagnosis and treatment of metastatic breast cancer, as suggested by [78].

The association between circulating miRNA and PCs was investigated via a correlation analysis. None of the principal components for all three normalization methods (within-normalization, z-score, and quantile) showed significant associations with the circulating miRNA. This may be due to the low number of patients (40% of the included patients) that reported the values for the circulating miRNA. However, circulating miRNA125b_5p was significantly different between the patients with grade 2 and 3 tumors. In particular, it was significantly lower in grade 3 breast cancer patients.

In conclusion, we provided a statistical framework that combines several approaches to generate a quantitatively robust and replicable radiomic signature, which may lead to more precise breast cancer prognosis and help clinicians in decision-making towards personalized medicine.

However, this study presents some limitations. Intra-reader agreement was not assessed for the segmentation of the lesions. This was mainly due to the fact that in this study we performed semi-automatic segmentation of breast lesions, which reduce segmentation time and inter- and intra-reader variability [79]. In fact, variation due to manual segmentation could be reduced or eliminated by semi-automated or completely automated segmentation algorithms. Moreover, the semi-automatic segmentation of breast lesions was carefully supervised by an expert radiologist with more than 10 years of experience. Moreover, in this study we only investigated first order and second order MRI radiomic features. In future studies, high-order radiomic features will be also considered in the framework of analysis. 

The main limitation of our study was that the patient sample that reported additional clinal information was relatively small and unbalanced. We developed the framework of analysis on 36 patients using their MRI and PET radiomic features. Then, we used a smaller sample of 27 patients to validate our approach. In addition, our study lacks further validation on a bigger cohort. As we are aware that the results generated on a small sample size cannot lead to a generalized conclusion, future studies will validate the results from this pilot study on a larger dataset. However, we applied several methodological steps to overcome this issue: (1) We used traditional machine learning which has less computational complexity than the more advanced ML algorithms and, therefore, less parameters to train reducing overfitting [80]. (2) Due to the low number of patients included in the study, no more than 1 feature for every 10 patients was employed to develop the models, as described in [55]. (3) We balanced the dataset with synthetic samples (SMOTE) [81]. (4) Repeated cross-validation (N = 100) was performed to guarantee the robustness of the results and to reduce overfitting [82]. Moreover, due to the limit of small sample, this radiomic framework may not stand for all subtypes of breast cancer, as we have only investigated specific subtypes (Luminal A and B). 

After this pilot study, which helped us to establish a robust workflow of analysis, upcoming work will include studies on a larger and more recent clinically annotated data set to verify and validate the results from this study. We will further assess the role of the MRI and PET phenotypes in combination with genomic and clinical information to improve the prediction power of the machine-learning-based models. At the same time, with this study we are notifying other researchers to implement a multivariate statistical framework of radiomic analysis for post-acquisition extraction and data processing, in order to ensure more robust findings.

## 5. Conclusions

This pilot study aimed to design a weighted statistical framework investigating the stability of the radiomic features of robustness and repeatability applied to MRI and PET analysis in general and evaluated the impact of normalization methods to generate a complex radiomic signature in breast cancer imaging, specifically. To conclude, the results from this study demonstrate that a combination of quantitative radiomic analysis via PCA shows potential as a means for high-throughput image-based phenotyping to automatically detect the grade, aggressiveness of the tumor, and breast cancer subtype. 

## Figures and Tables

**Table 1:** Patient characteristics.

Variables [N]	Number of Missing Patients	Median	Range [max–min]	Mean	SD
Age (years) [N = 27]	0	57	82–35	55.259	13.75
circulating miR-125b-5p [N = 22]	5	0.017	0.102–0.006	0.026	0.024
circulating miR-143-3p [N = 22]	5	0.009	0.061–0.002	0.018	0.018
circulating miR-145-5p [N = 22]	5	0.006	0.045–0.002	0.012	0.012
circulating miR_100_5p [N = 19]	8	0.010	0.051–0.004	0.017	0.014
circulating miR_23a_3p [N = 19]	8	0.155	0.438–0.039	0.19	0.13
ESTROGEN RECEPTOR STATUS (%) [N = 23]	4	90	99–0.5	75.87	32.289
PROGESTERONE RECEPTOR STATUS (%) [N = 24]	3	55	99–0.5	52.979	38.606
HER2 STATUS (%) [N = 10]	17	90	99–60	84.2	15.747
Ki-67 (%) [N = 24]	3	40	80–5	41.25	26.996
		**Number of Patients**		**Percentage (%)**	
Molecular subtype classification ER/PR/HER [N = 24]	3				
+/−/+		1		4.17	
+/+/−		13		54.17	
+/+/+		10		41.67	
Grading [N = 19]	8				
G2		11		57.89	
G3		8		42.11	

## Data Availability

The raw data supporting the conclusions of this article will be made available by the authors, without undue reservation.

## Supplementary Materials

The following supporting information can be downloaded at: https://www.mdpi.com/article/10.3390/diagnostics12020499/s1, Table S1: Description of Radiomic Features, Table S2: Modelling Performance to Detect Tumor Subtypes (Luminal A and B), Figure S1: 18F-FDG-PET/MRI scan image, Figure S2: Loading plots for three datasets, Figure S3: Comparison among models (LB: LogistBoost, LDA: Linear decision Analysis, and RF: Random Forest) used to classify tumor grade, Figure S4: PC3 quantile. Top 10 variable contribution, Figure S5: Comparison among models (LB: LogistBoost, LDA: Linear decision Analysis, and RF: Random Forest) used to classify high- and low-values of KI_67, Figure S6: Comparison among models (LB: LogistBoost, LDA: Linear decision Analysis, and RF: Random Forest) used to classify tumor subtype.
