# Supplementary material for: A Complex Radiomic Signature in Luminal Breast Cancer from a Weighted Statistical Framework: A Pilot Study
Source: Diagnostics (Basel). 2022 Feb 15;12(2):499. doi: 10.3390/diagnostics12020499 (PMC8871349; doi:10.3390/diagnostics12020499)
Supplement: Supplementary file 1 [file diagnostics-12-00499-s001.zip › diagnostics-1546320-supplementary.pdf]

Supplementary Materials

Table S1: Description of Radiomic Features.

<table>
<tr><th>Feature Classes</th><th>Imaging Techniques</th><th>Counts</th><th>Notes</th></tr>
<tr><td rowspan="4">First Order</td><td>ADC-MRI without contrast agent</td><td>5</td><td rowspan="4">Exhibits voxel alone based statistical features.</td></tr>
<tr><td>T2w-MRI (T2)</td><td>5</td></tr>
<tr><td>T1w-MRI post-contrast agent injection (T1C)</td><td>5</td></tr>
<tr><td>PET</td><td>5</td></tr>
<tr><td rowspan="2">Second Order</td><td>T2w-MRI (T2)</td><td>19</td><td rowspan="2">Describes surface texture.</td></tr>
<tr><td>T1w-MRI post-contrast agent injection (T1C)</td><td>19</td></tr>
<tr><td>SUV</td><td>PET</td><td>16</td><td>The standard uptake value (SUV), also known as standardized uptake value, is a simple way of determining activity in PET imaging,</td></tr>
<tr><td>Total</td><td></td><td>74</td><td></td></tr>
</table>

Table S2: Modelling Performance to Detect Tumor Subtypes (Luminal A and B)

**Modelling performance with PC6 (non-normalized)**					
	**Sensitivity**	**Specificity**	**Accuracy**	**Sensitivity+Specificity**	**AUC**
**LDA**	67%	49%	58%	1.16	59%
**RF**	89%	16%	52%	1.05	69%
**LogistBoost**	83%	24%	54%	1.07	54%
**Modelling performance with PC6 (z-score)**					
	**Sensitivity**	**Specificity**	**Accuracy**	**Sensitivity+Specificity**	**AUC**
**LDA**	67%	42%	55%	1.09	60%
**RF**	89%	17%	53%	1.06	75%
**LogistBoost**	90%	14%	52%	1.04	52%
**Modelling performance with PC3 (Quartile)**					
	**Sensitivity**	**Specificity**	**Accuracy**	**Sensitivity+Specificity**	**AUC**
**LDA**	80%	53%	66%	1.33	73%
**RF**	88%	24%	56%	1.12	75%
**LogistBoost**	79%	36%	57%	1.15	57%
**Modelling performance with PC4 (Quartile)**					
	**Sensitivity**	**Specificity**	**Accuracy**	**Sensitivity+Specificity**	**AUC**
**LDA**	78%	45%	62%	1.23	63%
**RF**	91%	26%	58%	1.17	76%
**LogistBoost**	83%	30%	56%	1.13	56%

AUC: Area Under the Curve; LDA: Linear Discriminant Analysis; RF: Random Forest.

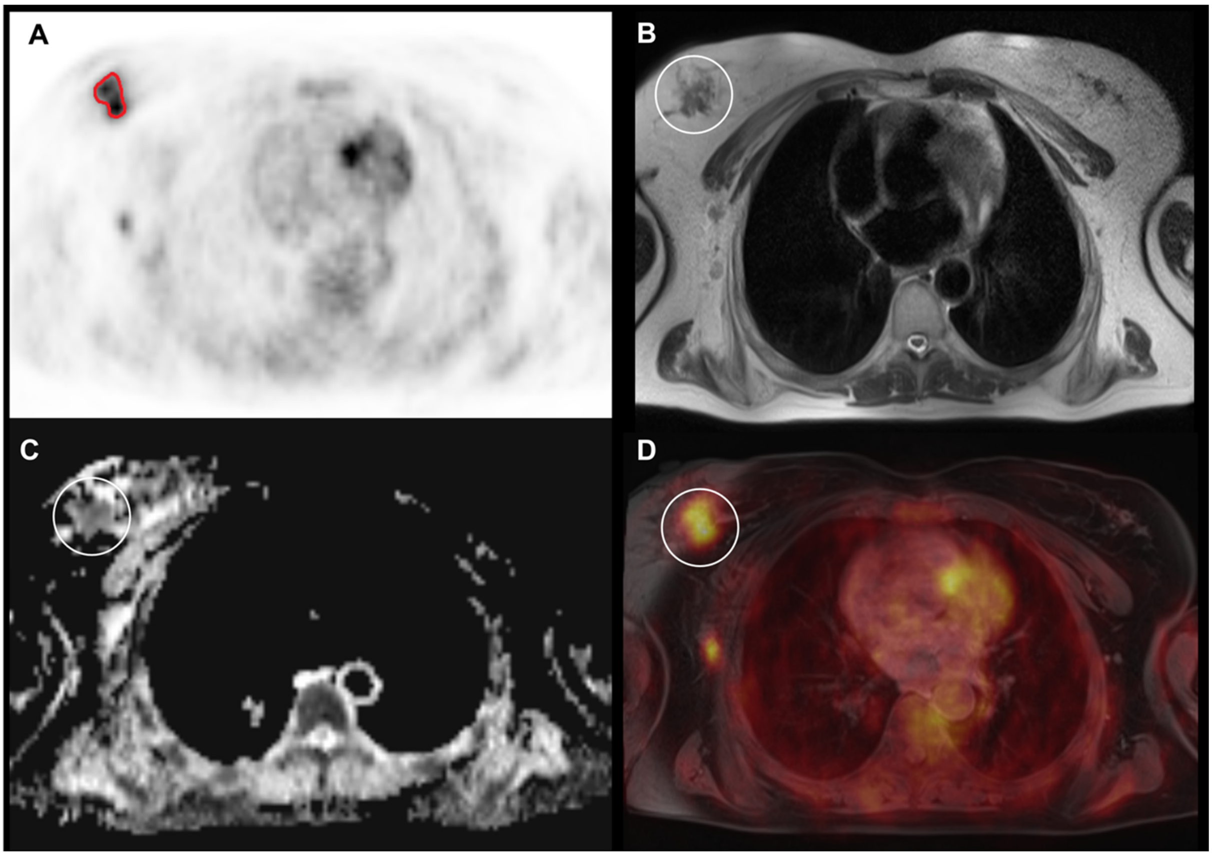

Supplementary Figure S1. 18F-FDG-PET/MRI scan Images of a 63-year-old patient with a breast heteroplastic process on the right breast. The white circle indicates the lesion: (A) PET image showing uptake after 60 minutes of a segmented lesion (red depicted); (B) Lesion revealed on T2 weighted image (T2WI) acquired on the axial plane; (C) hypointensity of the lesion on apparent diffusion coefficient (ADC) map; (D) 18F-FDG-PET/MRI fusion image.

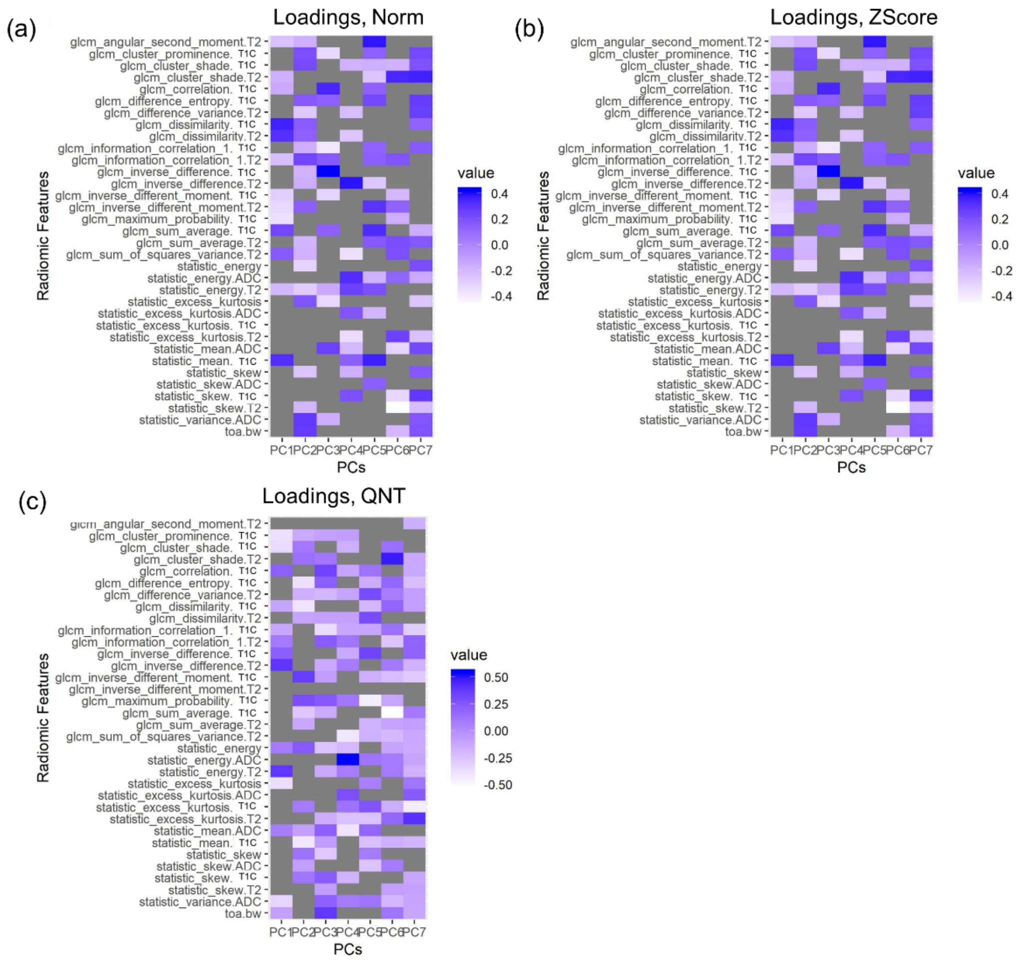

Supplementary Figure S2. Loadings Plots for 3 datasets. Highlighted boxes are above the 3rd quantile threshold. (a) Normalize only as ratio of malignant and healthy radiomic features; (b) Z-Score; (c) Quantile.

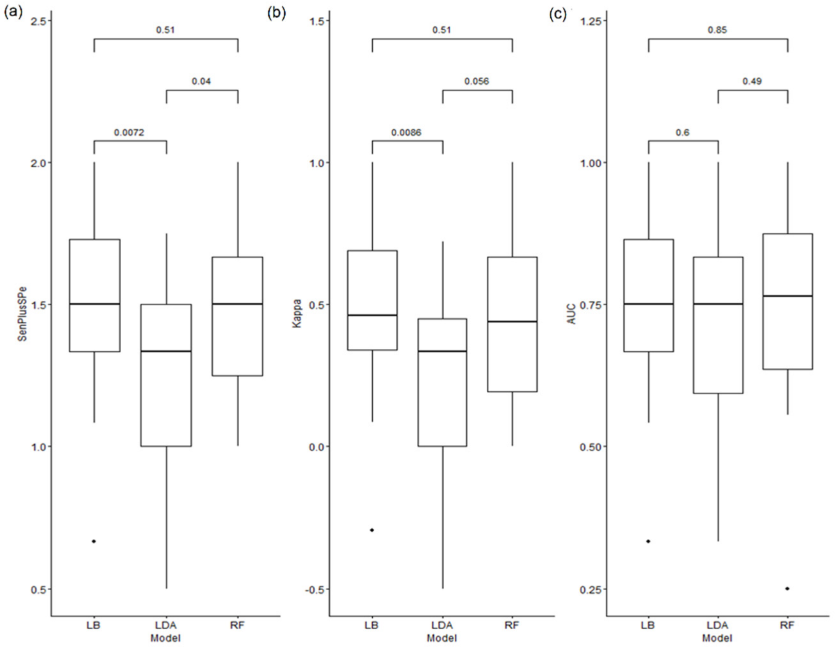

Supplementary Figure S3. Comparison among models (LB: LogistBoost, LDA: Linear decision Analysis, RF: Random Forest) used to classify tumor grade. (a) Boxplot of Sensitivity+Specificty value. (b) Boxplot of Cohen's Kappa. (c) Boxplot of AUC values.

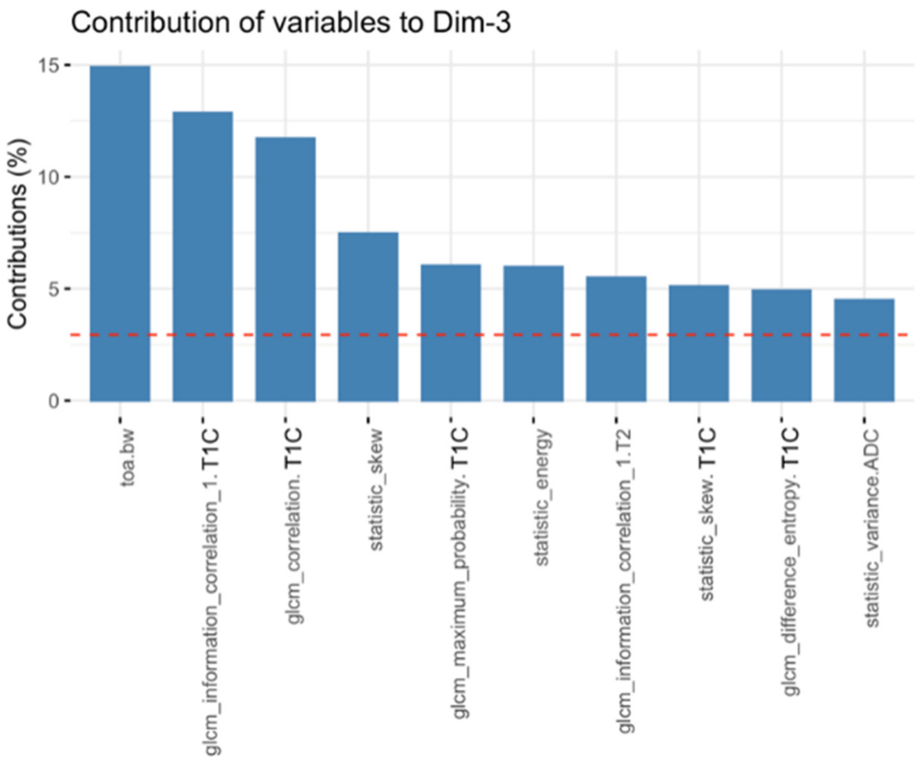

Supplementary Figure S4. PC3 Quantile. Top 10 variable contribution.

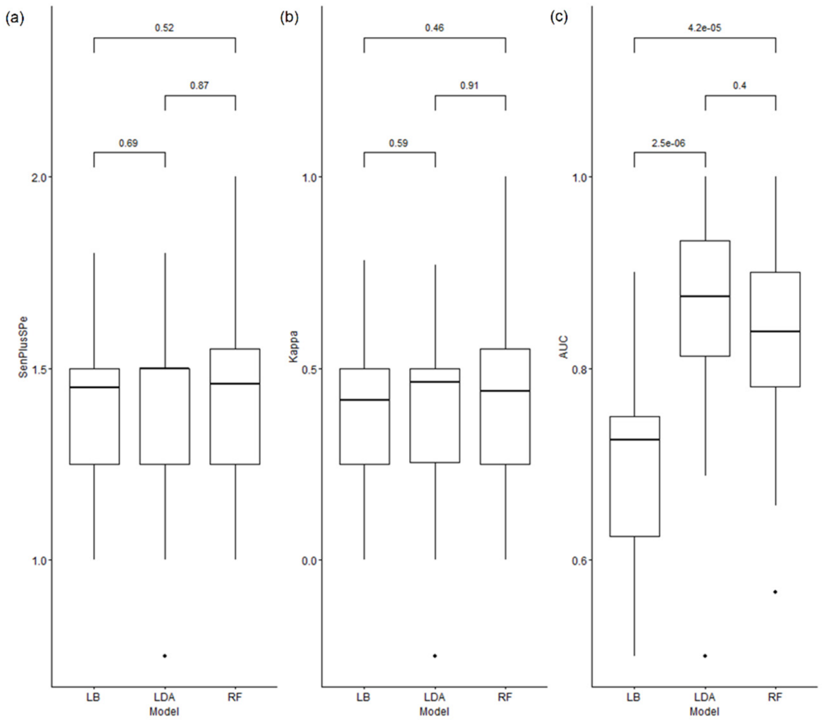

Supplementary Figure S5. Comparison among models (LB: LogistBoost, LDA: Linear decision Analysis, RF: Random Forest) used to classify high and low value of KI_67. (a) Boxplot of Sensitivity+Specificty value. (b) Boxplot of Cohen's Kappa. (c) Boxplot of AUC values.

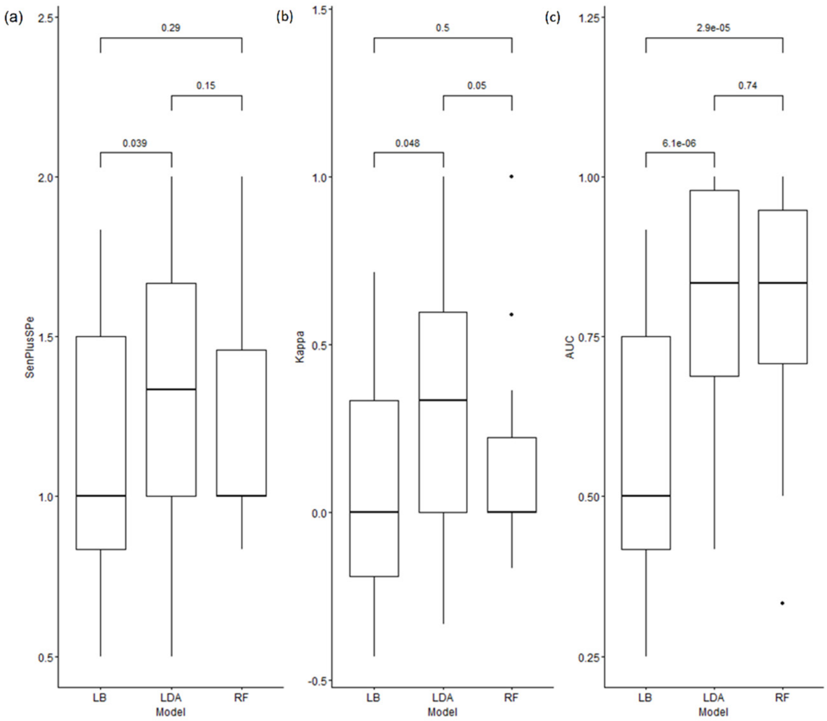

Supplementary Figure S6. Comparison among models (LB: LogistBoost, LDA: Linear decision Analysis, RF: Random Forest) used to classify tumor subtype. (a) Boxplot of Sensitivity+Specificty value. (b) Boxplot of Cohen's Kappa. (c) Boxplot of AUC values.
